# The Role of Apelin–APJ System in Diabetes and Obesity

**DOI:** 10.3389/fendo.2022.820002

**Published:** 2022-03-09

**Authors:** Cheng Li, Hongna Cheng, Binay Kumar Adhikari, Shudong Wang, Na Yang, Wenyun Liu, Jian Sun, Yonggang Wang

**Affiliations:** ^1^ Department of Cardiovascular Center, The First Hospital of Jilin University, Changchun, China; ^2^ Luohe Medical College, Luohe, China; ^3^ Department of Cardiology, Nepal Armed Police Force (APF) Hospital, Kathmandu, Nepal; ^4^ Department of Radiology, The First Hospital of Jilin University, Changchun, China

**Keywords:** apelin, diabetes, obesity, metabolic disorder, APJ

## Abstract

Nowadays, diabetes and obesity are two main health-threatening metabolic disorders in the world, which increase the risk for many chronic diseases. Apelin, a peptide hormone, exerts its effect by binding with angiotensin II protein J receptor (APJ) and is considered to be linked with diabetes and obesity. Apelin and its receptor are widely present in the body and are involved in many physiological processes, such as glucose and lipid metabolism, homeostasis, endocrine response to stress, and angiogenesis. In this review, we summarize the literatures on the role of the Apelin–APJ system in diabetes and obesity for a better understanding of the mechanism and function of apelin and its receptor in the pathophysiology of diseases that may contribute to the development of new therapies.

## 1 Introduction

The prevalence of metabolic disorders is increasing worldwide in recent years. Diabetes and obesity are two of the most common health-threatening metabolic disorders. It is reported that there are 3.7 million and 2.5 million people who die every year globally due to type 2 diabetes mellitus and obesity, respectively. Metabolic disorders contribute to the development of insulin resistance, obesity, cardiovascular complications, and, eventually, multi-organ dysfunction (1). Diabetes is a state of hyperglycemia and is characterized by insulin resistance and/or insulin secretion dysfunction. Sustained hyperglycemia and insulin hyposensitivity lead to the dysfunction of various tissues and organs. Obesity is an important risk factor that contributes to the development of type 2 diabetes by dysregulating several metabolic and adipose tissue-derived factors. Adipose tissues secrete adipokines, which are involved in glucose and lipid metabolism, neuroendocrine function, insulin sensitivity, and other physiological processes. Therefore, an effective and novel therapeutic recommendation is needed to curb the incidence, morbidity, and mortality caused by metabolic diseases.

Apelin is a regulatory peptide as well as a ligand for G-protein-coupled receptor (APJ). It is widely present in the body, including in peripheral tissues and the central nervous system. There is a growing interest on the apelin–APJ system during the past decade due to its potential role in several physiological processes, such as homeostasis, body fluid management, cell proliferation, and energy metabolism (2–4). Apelin is produced and secreted by adipocytes; hence, it is called adipokine. Several studies have reported that the increased plasma apelin is related to metabolic pathologies. Evidences are in favor of the regulatory role of the apelin–APJ system in glucose and lipid metabolism. The present review summarizes the current knowledge and literatures on the regulatory role of apelin, with emphasis on the regulation of glucose and lipid metabolism, that may provide novel therapeutic targets.

## 2 Apelin–APJ Receptor System Discovery and Development

### 2.1 Apelin and Its Receptor APJ

**Figure 1:**
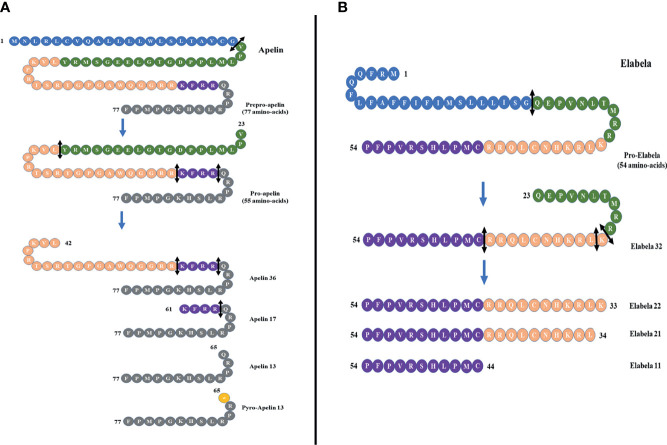
**(A)** Different forms of apelin. The proteolysis of preproapelin at specific cleavage sites (double-headed arrows, black) leads to three fragments of 36, 17, and 13 amino acids named apelin-36, apelin-17, and apelin-13, respectively. The N-terminal glutamine residue of apelin-13 is pyroglutamylated, which produces the pyroglutamyl form of apelin-13 ([Pyr1]-apelin-13). **(B)** The proteolysis of proelabela at specific cleavage sites (double-headed arrows, black) leads to four fragments of 32, 22, 21, and 11 amino acids named elabela-32, elabela-22, elabela-21, and elabela-11, respectively.

In 1993, O’Dowd et al. isolated a special human G protein-coupled receptor and named it “APJ”. It contains 380 amino acids and shares 54% sequence with the human angiotensin II receptor type 1 in the transmembrane (5). Subsequently, APJ receptor was also isolated in amphibians and rodents (6, 7). The APJ gene is located on chromosome 11 (11q12) without any intron in the coding region. However, angiotensin II does not interact with APJ receptor, and there was no APJ ligand identified until 1998, when apelin, a APJ endogenous ligand, was isolated by Tatemoto et al. from the bovine stomach (8). The apelin gene is located on Xq25-q26.3 chromosome. It encodes a 77-amino-acid prepropeptide that contains a secretory signal sequence and can be cleaved into different active forms such as apelin-36, apelin-17, apelin-13, and apelin-12 (8) (**Figure 1A**). Apelin-36 is the most widely expressed form, while apelin-13 is more potent and more abundant in the circulation (9). It has been demonstrated that there is a high sequence homology among human, bovine, and rodent preproapelin, particularly the last 22 C-terminal amino acids (8, 10). All isoforms of apelin could bind to APJ, but they have a different biological potency. Different organs have different apelin isoforms.

Apelin and APJ are widely expressed in many tissues and organs, including the brain, heart, lung, liver, kidney, gastrointestinal tract, endothelium and adipose tissues (11). Apelin-12 possesses a high affinity for APJ receptor, with cardioprotective properties of increasing the myocardial contractility and reducing the mean arterial pressure (12). Apelin-13 is the main neuroprotective peptide, with the highest abundancy in the plasma (13, 14). Previous research reported that apelin-13 participates in vasculopathy, energy metabolism, and humoral homeostasis (13, 15, 16). There was also a study that discovered the downregulation of apelin-13 in aging. The knockout of apelin-13 and APJ gene could accelerate aging in a mouse model, while the upregulation of apelin-13 restores vitality, a response to the stimuli and circadian rhythm (17). Glutamine cyclase can catalyze the translated N-terminal glutamine residues of apelin-13 to modify pyroglutamidation and produce the pyroglutamide form of apelin-13, called [pyr^1^]-apelin-13. It can prevent apelin-13 from degradation by exopeptidase and exert long-term biological effects (4). Therefore, [pyr^1^]-apelin-13 is considered a physiological ligand for APJ due to its higher anti-degradation properties (18). Apelin-17 was found to be the most potent inducer of APJ internalization, and the removal of a single amino acid at the C-terminus can abolish this process (19). Apelin-36 is mainly expressed in the lung, testis, and uterus (20). Apelin-13 and apelin-36 have been considered the most active isoforms with the greatest effect on the cardiovascular system (21). Apelin-13 and apelin-36 have different intracellular trafficking of APJ due to different receptor binding affinities. In addition, the shorter apelin isoform seems to be the more potent activator of APJ than apelin-36 (22). It is worth noting that APJ knockout mice showed a disrupted cardiac development with rudimentary to absent heart resulting in prenatal mortality (23). On the contrary, apelin knockout mice had normal heart development (24). It indicates that there may be other ligands for APJ.

### 2.2 Novel APJ Ligand—Elabela/Toddler

Apelin was considered to be the only APJ ligand until 2013, when two different research groups discovered a second endogenous ligand for the apelin receptor in zebrafish (*Danio rerio*) during embryonic development, and it was named Elabela (“epiboly late because endoderm late”) by Chng et al. and Toddler (referring to the loss of motogen properties when deleted) and by Pauli et al. (25, 26). Subsequently, the cDNA encoding Elabela was discovered in vertebrates and mammals (27). Recently, Elabela was found to be restrictedly expressed in human pluripotent stem cells and renal tissues. Elabela is a peptide of 54 amino acids with a secretory signal containing 32 amino acids (25) (**Figure 1B**). This precursor is cleaved to shorter sequences of 32, 21, and 11 amino acids (28) (**Figure 1B**), and all these short sequences can activate APJ as well as can be blocked by apelin receptor antagonists.

The discovery of this new ligand created several exciting possibilities. Evidences revealed the role of Elabela in homeostasis, cell signaling, energy metabolism, and cell aging. Serum Elabela is associated with several diseases, such as diabetes (29), myocardial infarction (30), hypertension (31), and kidney disease (32). Notably, Elabela level is also related to the prognosis of these diseases. It is reported that Elabela can ameliorate apoptosis *via* regulating the mitochondrial function (33). Recently, Elabela has been demonstrated to decrease kidney injury (34), and serum Elabela level in type 2 diabetes patients is closely related to the severity of renal injury (35). In addition, Elabela has been reported to improve endothelial cell function *via* the PI3K/Akt signaling pathway (36). Some literatures also showed that Elabela is a potent regulator of adipose cell metabolism. However, the effect of Elabela is still unclear, and further studies are required to clarify its role and significance.

## 3 Effect of Apelin–APJ System on Diabetes

**Figure 2:**
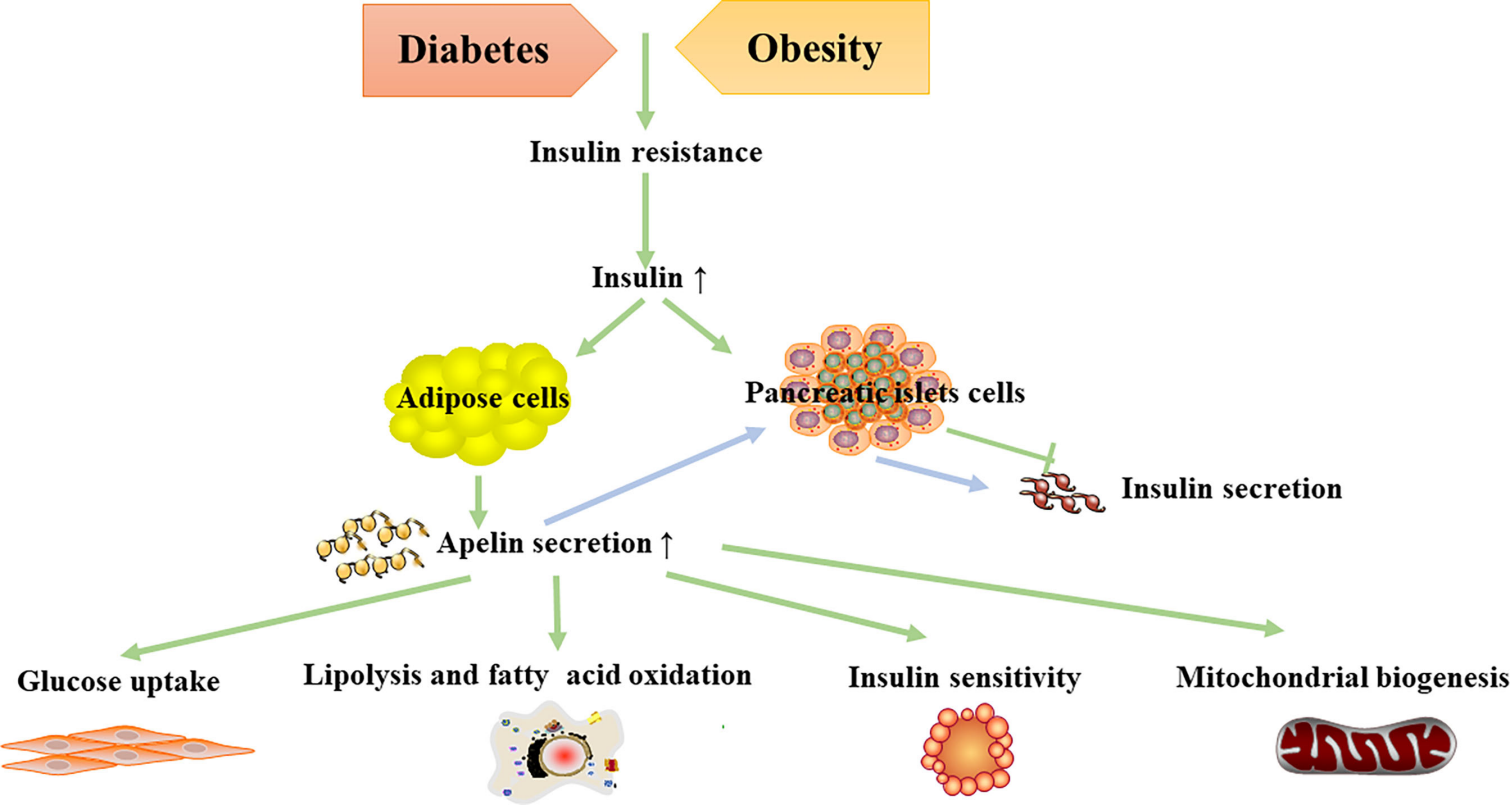
Metabolic effects of apelin in diabetes and obesity.

Nowadays, apelin is believed to assist in regulating glucose metabolism, and the apelin–APJ system has been demonstrated to be related with diabetes mellitus and diabetic complications. Apelin stimulates glucose uptake, increases insulin sensitivity, and regulates lipolysis and fatty acid oxidation (**Figure 2**). Diabetes-related diseases can be improved by apelin administration. Therefore, the apelin–APJ system is a potential therapeutic target in diabetes and its complications.

### 3.1 Apelin and Glucose Metabolism

Apelin is predominantly expressed in the beta and alpha cells of pancreatic islets, and APJ receptor is expressed in acinar cells and pancreatic ductal cells (37). Insulin is considered the prime regulator of apelin that stimulates its synthesis and release. Apelin is also affected by hypoxia and adiposity. In normal and insulin-resistant mice, apelin was noted to promote peripheral glucose uptake (38). Exogenous apelin administration was found to enhance glucose metabolism (39). Moreover, apelin-induced glucose uptake was also detected in isolated normal adipocytes and type 2 diabetic adipocytes (39, 40). These results indicate that apelin might act as an exogenous insulin sensitizer under high insulinemia.

However, in the beta cells of type 2 diabetes (T2D) db/db mice, the apelin level was detected to be upregulated. The administration of apelin-36 can inhibit glucose-stimulated insulin secretion (41). It is reported that apelin inhibits insulin secretion *via* stimulating the degradation of cAMP due to the activation of phosphodiesterase 3B activity, which subsequently results in the impairment of glucose elimination (42). Sorhede et al. found that insulin binds its receptor on adipocytes and promotes the expression of apelin. It provides negative feedback for insulin secretion (41). On the contrary, Gao Z et al. reported that the administration of apelin-13 significantly reduces blood glucose and increases serum insulin level (43). Besides these, long-term apelin administration significantly improves pancreatic islet mass and insulin level in diabetes (44). These effects were related to the upregulation of PERK-IRE1a-CHOP signaling and the deactivation of AKT, ERK, and AMPK in the pancreas of diabetic mice (44, 45).

### 3.2 Apelin–APJ System and Diabetic Complications

**Figure 3:**
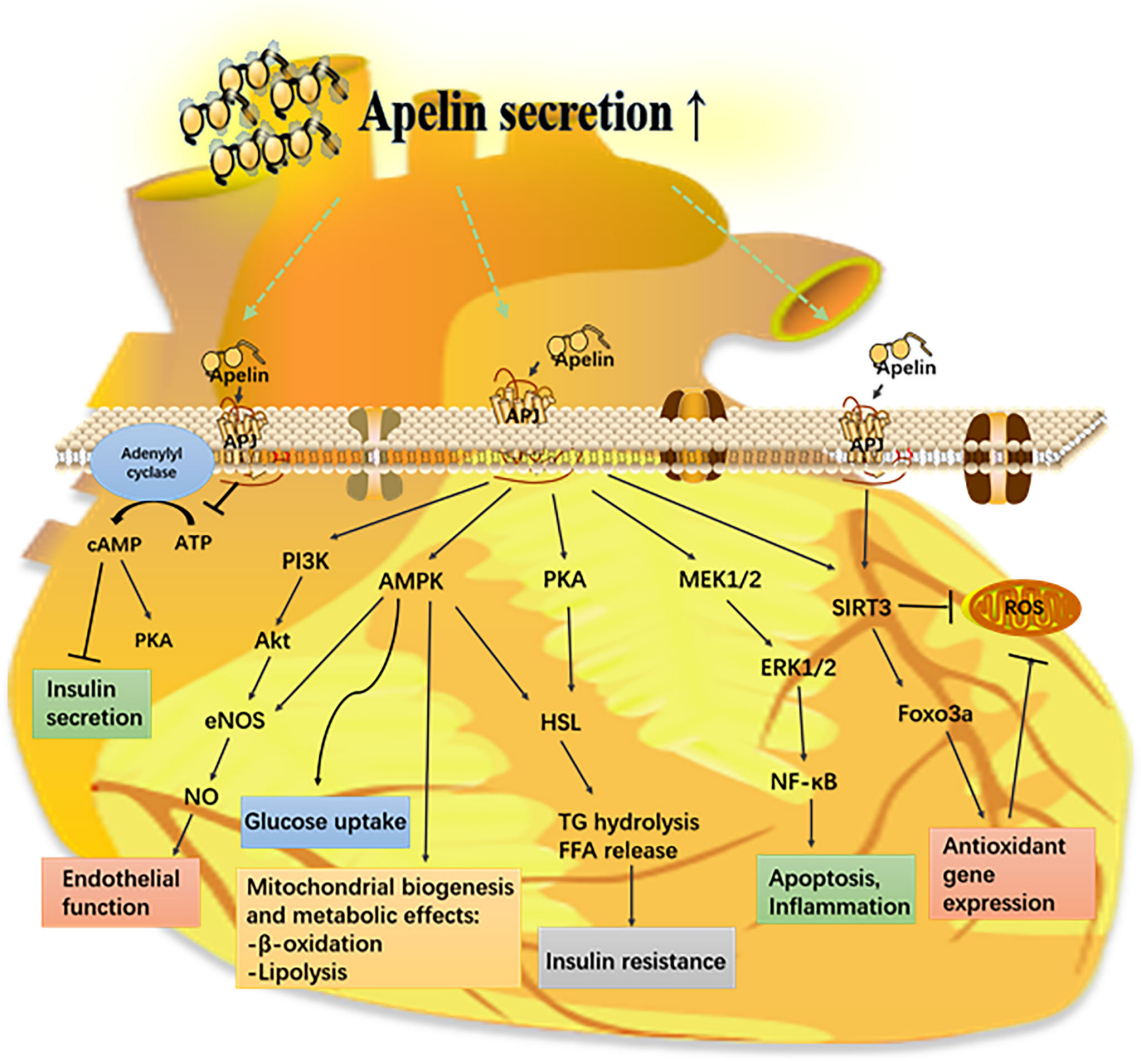
Mechanism of the apelin–APJ system in diabetes and its complications. Apelin activates its receptor (APJ) and triggers various signaling pathways that have a protective effect on different organs from metabolic diseases. AMPK, AMP-mediated protein kinase; eNOS, endothelial nitric oxide synthase; ERK1/2, extracellular-regulated kinases 1/2; FFA, free fatty acid; Foxo3a, forkhead box protein O 3a; HSL, hormone sensitive lipase; PI3K, phosphoinositide 3-kinase; PKC, protein kinase C; ROS, reactive oxygen species; SIRT3, sirtuin 3.

Apelin has a significant role in regulating insulin secretion, oxidative stress, apoptosis, and angiogenesis; hence, it is involved in the pathogenesis of diabetic complications (**Figure 3**).

#### 3.2.1 Cardiovascular Diseases

Both cardiomyocytes and microvascular dysfunction are critical in inducing diabetic cardiomyopathy. Apelin peptides are considered the most potent endogenous positive inotropic agent in the myocardium, and apelin-13, as the predominant apelin isoform in human myocardium, has been reported to play a crucial role in myocardial contraction, vascular relaxation, blood pressure regulation, and insulin sensitivity (46–48). Long-term apelin-13 administration can prevent pancreatic beta cell loss or dysfunction in type 2 diabetic rat models and reduce myocardial fatty acid uptake and oxidation through inhibiting the PPAR-α receptor (49). In addition, the upregulation of apelin inhibits apoptosis and oxidative stress *via* the PI3K and p38-MAPK pathways, resulting in protection from ischemia–reperfusion injury in diabetic myocardium (50).

SIRT3, a member of sirtuins family, is considered an essential transcription factor in the apelin-induced protection of diabetic cardiomyopathy. Previous studies showed that apelin increases SIRT3 expression, improves cardiac function, and ameliorates diabetic cardiomyopathy. Treatment with apelin increases the myocardial vascular density *via* upregulating SIRT3 (51) and VEGF/VEGFR2 expression (52), which also ameliorates diabetic cardiomyopathy and improves the echocardiography parameters of cardiac function. In post-myocardial infarction diabetic mice, the overexpression of apelin markedly upregulates SIRT3 and angiogenic growth factor expression (51, 53). Apelin gene therapy increases the expression of angiogenic growth factors and angiogenesis in endothelial progenitor cells, but these effects do not occur in SIRT3-knockout endothelial progenitor cells (51). Moreover, apelin treatment dramatically increases autophagy *via* upregulating SIRT3 and downregulating NF-κB-p65 expression in the myocardium of STZ-induced mice (53). Icariin has a cardioprotective effect in high-glucose-treated cardiomyocytes *via* upregulating apelin and SIRT3 expression, which can reverse diabetes-induced mitochondrial dysfunction; however, it did not affect the activity of SIRT3 in apelin silence samples (54). In addition, our previous research showed that Elabela can regulate the SIRT3-mediated inhibition of oxidative stress through Foxo3a deacetylation and prevent diabetic-induced myocardial injury (55). These results suggest that the apelin/SIRT3 signal pathway may be used as a novel therapeutic strategy for diabetes-related cardiovascular diseases.

Macrovascular injury is another important aspect of diabetes-related complications. Impairment of vascular contraction and dilatation is the hallmark of endothelial dysfunction, which is responsible for diabetic vascular pathophysiology. A previous research reported that the apelin–APJ system reduces vasodilatation and increases vasoconstriction in insulin resistance-related disorders, such as diabetes and cardiovascular dysfunction (56, 57). Zhong and his colleagues found that apelin plays a crucial role in vascular tone maintenance in diabetic mice by counteracting the vasoconstricting action of Ang II and potentiating the release of NO through the activation of the Akt-eNOS phosphorylation pathway (57). There is a high expression of apelin in the aorta of type 2 diabetes rat models (58). *In vitro*, apelin-13 can inhibit high-glucose-induced cell proliferation, migration, and invasion of aortic smooth muscle cells. Apelin-13 can also effectively attenuate high-glucose-induced calcification and dramatically suppress high-glucose-induced DNA injury through inhibiting reactive oxygen species (59).

In children with type 1 diabetes mellitus, the serum apelin level has a positive correlation with the carotid intima–media thickness, which indicates that serum apelin might be used as a predicting factor for atherosclerosis (60). However, in patients with type 2 diabetes mellitus, although the apelin level was lower in patients with diabetic complications than in patients without complications, there was no significant difference (61). In another clinical study, patients with hypertension and/or type 2 diabetes have a lower serum apelin level, along with cardiac remodeling, and primarily concentric left ventricle hypertrophy; moreover, there is a negative correlation of apelin with cardiac structural parameters such as left ventricle remodeling and left atrial size (62). Another clinical research showed that physical training increases the apelin level, with a reduction of low-density lipoprotein cholesterol (LDL-C) and hsCRP levels, and insulin resistance, resulting in the decreased progression of the carotid intima–media thickness in patients with type 2 diabetes (63).

#### 3.2.2 Diabetic Nephropathy

Diabetic nephropathy (DN) is characterized by glomerular, tubular, and tubulointerstitial injury caused by hyperglycemic status. The early stage of DN presents as glomerular hypertrophy and thickening of glomerular basement membrane. With the development of this disease, high glomerular filtration leads to proteinuria, eventually resulting in end-stage renal disease (64). APJ is expressed in the glomeruli and blood vessels of kidney, while apelin is expressed in renal vascular endothelial cells and is highly expressed in the inner stripe of the outer medulla (65). A previous study showed that serum apelin and its receptor APJ level were increased in DN patients, and a higher apelin and APJ level promoted the formation of blood vessels and induced the proliferation of glomerular capillaries, thus accelerating the development of DN (66). However, there are conflicting reports about the apelin level in diabetes. It was increased in some research (67, 68), while it was decreased in other research (69). It is speculated that a lower apelin level may be caused by apelin-regulated insulin sensitivity, which stimulates glucose utilization and enhances brown adipogenesis (70). In high-glucose-medium-cultured podocytes, APJ mRNA is upregulated when compared to the normal condition (71). It is reported that renal APJ expression is decreased in diabetic mice, but after apelin treatment, it is increased (72). Recent studies also found that apelin can control the reduction of podocyte proteasome activity *via* inducing endoplasmic reticulum stress and podocyte dysfunction through the regulation of the ERK, Akt, and mTOR-related pathways. These all contributed to the development of DN (73, 74).

Since Elabela is predominantly expressed in the kidney, the relationship between serum Elabela level and DN is also reported. Zhang et al. reported that the serum Elabela level was lower in T2D patients with albuminuria. Particularly, the serum Elabela level decreased progressively with the progression of DN; moreover, the serum Elabela level has a negative correlation with blood pressure, retinopathy, serum creatinine, and urinary albumin/creatinine ratio and has a positive correlation with the estimated glomerular filtration rate (29). A similar result was also confirmed by Erhan et al. They found that the Elabela level was higher in healthy individuals when compared with diabetic patients without microalbuminuria and even higher in diabetic patients without microalbuminuria when compared to patients with advanced albuminuria (35). These results suggest that Elabela may be an important clinical prognostic marker.

However, exogenous apelin and Elabela have been demonstrated to slow down the progression of DN. Apelin-13, the most active subtype of apelin, was paid more attention in DN. It was reported that Apelin-13 administration can reduce proteinuria, glomerular hypertrophy, mesangial expansion, and renal inflammation in type 1 and type 2 diabetic models (71, 75, 76). Hong et al. reported that apelin-13 treatment inhibits the hyperglycemia-induced elevation of inflammatory factors and histone hyperacetylation by upregulating histone deacetylase 1 (76). It was also reported that apelin-13 administration regresses DN by promoting the production of nitric oxide and alleviating renal fibrosis (73). Besides these, exogenous apelin inhibits the epithelial–mesenchymal transition of podocytes in diabetic mice *via* decreasing the expression of the immunoproteasome subunit β5i (77). Zhang Y et al. reported that the protective effect of Elabela in type 1 diabetes-induced podocyte injury might be related to the activation of the PI3K/Akt/mTOR pathway (78). However, further research is required to understand the mechanism of the apelin–APJ system in DN progression.

#### 3.2.3 Vascular Effects, Endothelial Dysfunction, and Angiogenesis

Apelin has both vasodilation and vasoconstriction effects because the apelin–APJ system in the endothelium and smooth muscle cells exert an opposite action in regulating the vascular tone (2, 79). When apelin binds to endothelial APJ, it promotes the secretion of endothelium-derived relaxing factors, such as nitric oxide (NO) and prostacyclin, resulting in vasodilation (80–82). When apelin binds to smooth muscle APJ, it causes vasocontraction (83, 84). In a diabetic animal model, APJ expression was lower in the aorta and renal arteries, and Ang II-induced contractile responses were enhanced, but apelin administration reversed the abnormal vascular response to Ang II (57, 85).

The vascular endothelium behaves as an autocrine as well as paracrine organ in regulating vascular homeostasis. When it is impaired by hyperglycemia, vasoconstriction may occur and be accompanied with leukocyte adherence, platelet activation, oxidative stress, inflammation, thrombosis, and atherosclerosis (86). The apelin–APJ system is involved in diabetes-induced endothelial dysfunction and angiogenesis (**Figure 3**). It is reported that apelin might decrease apoptosis and the expression of adhesion molecules and increase proliferation and angiogenesis *via* APJ-activated NF-κB pathways, finally resulting in the protection of diabetes-induced endothelial dysfunction (87). In high-glucose-treated microvascular endothelial cells, apelin-12 suppressed apoptosis, inflammation, and oxidative stress *via* regulating the p-JNK and p-p38 pathways (88). Methylglyoxal, as a glycolytic metabolite, has been demonstrated to have a greater potential to stimulate endothelial dysfunction than glucose itself (89, 90). Previous studies have shown the impairment of endothelium-dependent vasorelaxation by methylglyoxal, mostly mediated by oxidative stress (90). Kim Sujin et al. demonstrated that apelin-13 can inhibit methylglyoxal-induced endothelial apoptosis and endoplasmic reticulum stress through the AMPK pathway (91). In diabetic Lepr^db/db^ mice, apelin-36 restores the altered aortic vascular responsiveness to acetylcholine and Ang II by potentiating the phosphorylation of Akt and eNOS (57). In high-fat-diet-treated Apoe^-/-^ mice, the loss of apelin results in exacerbation of atherosclerosis, while apelin administration leads to a significant regression of atherosclerosis, which may be related to the activation of the nitric oxide pathway and inhibition of Ang II signaling (92).

In addition, apelin is confirmed as a potent angiogenic factor, especially in retinal endothelium. It is reported that the serum apelin-13 level has a positive correlation with proliferative diabetic retinopathy, which is unrelated with VEGF (93). Research by Yasir et al. and Wu et al. concluded similar results (94, 95). Li Yang et al. found that apelin can induce the proliferation, migration, and expression of the cytoskeleton and tight junction protein through the PI3K/Akt and MAPK/ERK pathways in human retinal pigment epithelial cells (96). Furthermore, in post-myocardial infarction diabetic mice, the overexpression of apelin mobilizes endothelial progenitor cells to ischemic regions and contributes to angiogenesis (51). Therefore, apelin may be a promising therapeutic target for diabetic angiogenesis-related diseases.

#### 3.2.4 Central Nervous System

As mentioned above, apelin and APJ are widely expressed in the human central nervous system, more in the oligodendrocytes and neurons and less in astrocytes (97). The apelin–APJ system is also observed in the pituitary gland, indicating a role in the control of the hypothalamic–pituitary–adrenal axis (HPA). A study shows that APJ acts as a neuromodulator in modifying the HPA axis activity after acute stress stimuli (98). It was also reported that APJ-deficient mice, under hypoglycemic stress, had decreased ACTH release, confirming the role of central apelin in neuroendocrine functions. Recent studies have suggested that central apelin is involved in the transition from normal to diabetic state (99). These findings indicated that central apelin may control glucose release and glucose metabolism. Anne et al. found that the intracerebroventricular injection of apelin increases fasting blood sugar (99), which was related with the over-activation of the sympathetic nervous system, followed by liver glycogenolysis and gluconeogenesis (100). The over-expression of hypothalamic apelin was observed in obesity and diabetes (101). These results indicate that the apelin–APJ system in the central nervous system may be a new target for controlling glucose metabolism.

## 4 Effect of the Apelin–APJ System on Obesity

Adipose tissue plays a central role in lipid and glucose metabolism (**Figure 2**) and is now considered a major endocrine organ. Apelin and its ligand are expressed in adipose tissue. Apelin is secreted by adipose tissue; thus, it is also called adipokine (102).

### 4.1 Apelin and Lipid Metabolism

Apelin, as an adipokine, is considered a crucial modulator of lipid metabolism. The plasma apelin level is lower in patients with elevated LDL-C when compared with the healthy controls (103). LDL-C reduction by statins is accompanied with an increase in serum apelin level in dyslipidemic patients (104). Apelin deficiency mice display increased adiposity and elevated circulating free fatty acids (105), whereas transgenic mice with over-expressed apelin is resistant to obesity (106). On the contrary, it was reported that cardiac apelin and APJ expression and serum apelin level were increased in obese rats, and downregulation of apelin and APJ expression alleviated insulin resistance and inflammation (107).

Chronic apelin treatment reduces fat mass and increases muscle oxidative capacity as well as mitochondrial biogenesis (108). Chronic apelin treatment showed a decrease of hepatic steatosis by reducing *de novo* lipogenesis in insulin-resistant mice (109). Apelin-13 can improve glucose and lipid metabolism and reduce the damage caused by oxidative stress and inflammation *via* the PI3K/Akt pathway in gestational diabetes mellitus mouse (110). Another animal experiment also showed that apelin-13 regulates the expression of PPARγto inhibit adipogenic differentiation and regulates the expression of perilipin to promote lipolysis, thereby reducing obesity (110). In a rat model of type 2 diabetes with a high-fat diet, apelin-13 reduces serum total cholesterol, triglyceride, and LDL-C and increases high-density lipoprotein cholesterol (HDL-C) (111). Moreover, apelin-13 promotes cholesterol efflux and decreases foam cell formation, which indicates its potential anti-atherogenic effect (112, 113). Besides these, Chun et al. found that apelin-13 administration abrogates angiotensin II-induced atherosclerosis in ApoE^-/-^ mice through promoting NO production and inhibiting the angiotensin II intracellular pathway (92). Moreover, apelin-13 greatly ameliorates plaque stability *via* increasing the intraplaque collagen content and reducing the MMP-9 expression (114). In addition, apelin administration effectively diminishes the LDL-C/HDL-C ratio and the atherogenic index in Wistar rats with hypothyroidism (115). Hashimoto et al. found that the apelin–APJ system is the mediator of oxidative stress-linked atherosclerosis in blood vessels (116).

### 4.2 Apelin, Insulin Resistance, and Obesity

In obesity, adipocytes release more free fatty acids, which contribute to the development of insulin resistance. It is observed that insulin resistant individuals have a higher level of circulating free fatty acids (117). Apelin, as an adipokine, is upregulated in obesity. In clinical and experimental studies, serum apelin level or its adipose tissue expression is increased in obesity and insulin resistance status (68, 118). Patrick Yue et al. reported that apelin knockout significantly increases the serum concentration of glycerol, leptin, and free fatty acids, while exogenous apelin administration decreases these compounds (105). It is reported that supplementary apelin improves *in vitro* insulinotropic activity, glucose uptake by adipocyte, glucose elimination, and insulin release in obese mice (119). Bolus intravenous apelin administration improves glucose tolerance and insulin sensitivity during a hyperinsulinemic–euglycemic clamp in obese and insulin-resistant mice (38), which indicates that exogenous apelin is efficient despite an elevated plasma apelin level. After 28 days of apelin therapy, a marked improvement in insulin sensitivity and decrease in body fat have been observed in obese and insulin-resistant mice (108). However, administration for 4 weeks of Fc-apelin-13 (apelin-13 fused with IgG Fc fragment) in obese mice significantly improves glucose tolerance, stroke volume, and cardiac output, while it decreases cardiac and hepatic fibrosis; but it does not affect food intake and body weight (120). When a chow diet was fed to 8-week-old apelin^-/-^ mice, insulin level was significantly increased, and plasma adiponectin concentration and glucose intolerance were decreased. In addition, these mice also showed increased abdominal and epididymal fat without a difference in body weight (105). The apelin^-/-^ mice were more glucose and insulin intolerant when they were fed with a high-fat diet and high-sucrose drinking water (121). Stable apelin-13 peptide analogues have shown promising short-term antidiabetic effects in mice with diet-induced obesity and diabetes (122).

Human studies revealed that Pyr1-apelin-13 injection in obese patients improves insulin sensitivity (123). However, a 6.5-year follow-up in overweight or obese children showed that the apelin level decreased significantly during pubertal development (124). In addition, Cavallo MG et al. reported that obese patients with T2D had a significantly higher apelin level than non-diabetic obese patients (125). Dayem et al. found that the apelin level has no influence on body mass index in diabetic patients (126). These results indicate that increased apelin level is directly associated with accompanying diabetes rather than obesity itself.

## 5 Drugs Targeting the Apelin–APJ System

The three-dimensional structure of APJ receptor is first reported by Langelaan and his colleagues (127). They presented a structure of the N-terminus and the first transmembrane segment of APJ (residues 1–55, AR55) that was comprised of residues essential for apelin binding in dodecyl phosphocholine micelles, which provided a new insight into the development of drugs. In recent years, some agonists and antagonists of APJ receptor have been discovered and synthesized, which have presented obvious therapeutic effects in animal models and patients. There are two natural endogenous ligands for APJ receptor, namely, apelin and Elabela. As mentioned above, apelin can be cleaved into different active forms such as apelin-36, apelin-17, apelin-13, apelin-12, and Pyr-apelin-13. Elabela, another APJ endogenous ligand, is also a strong agonist for APJ. In addition, there are some biosynthetic compounds targeting APJ receptor. E339-3D6 is the first nonpeptide APJ receptor agonist which was synthesized by Iturrioz et al. in 2010 (128). Then, other APJ receptor agonists were also synthesized, such as MM07, ML233, and CMF-019 (129–131). Meanwhile, several research on APJ antagonists were also conducted. Lee et al. generated an analog of apelin-13, called apelin-13 F13A, and found that it can block the hypotensive effect of apelin-13 (132). In addition, ALX40-4C is the first peptide antagonist for APJ receptor, which is a polypeptide comprised of nine arginine residues (133). ML221 is identified as the first non-peptide antagonist isoform of APJ, which blocks apelin-13-mediated cAMP production and β-arrestin recruitment for APJ (134). Further studies should focus on the beneficial effect of the compounds that target APJ and explain the therapeutic effect of novel synthetic ligands for APJ receptor in diabetes and obesity.

## 6 Conclusion

The apelin–APJ system is considered an emerging target with potential therapeutic properties in diabetes and obesity. Current literatures have suggested that apelin administration has effective protection in diabetic and/or obese mice. Current studies have shown a difference in serum apelin level between diabetic and/or obese patients and the control group, which supports the role of apelin in diabetes and obesity development and emphasizes the use of apelin as a clinical marker in diabetes and obesity. Despite numerous clinical and experimental studies clearly supporting the physiological and pathophysiological roles of the apelin–APJ system in glucose and lipid metabolism, the role of apelin in this complicated system is not yet fully elucidated, including relevant signaling pathways and biological adverse effects, especially in human. In the future, it will be important to identify the mechanism of action of apelin, such as the role of new receptors or regulating ligands or specific Elabela/apelin isoforms.

The apelin–APJ signaling system has emerged as an important biomarker and a novel therapeutic target against the development of metabolic diseases, especially diabetes and obesity. In this review, we summarized the literatures on the role of the apelin–APJ system in diabetes and obesity, and we hope for further research that will establish their role as a new diagnostic marker or therapeutic agent in diabetes and obesity.

## Author Contributions

CL reviewed the literature and drafted this review. HC reviewed the literature and corrected the figures. BA, SW, and NY reviewed the literature, gave critical comments, and revised the manuscript. WL gave critical comments and revised the manuscript. JS and YW reviewed the literature, gave critical comments, revised the manuscript, and took charge of project supervision and administration. All authors contributed to the article and approved the submitted version.

## Funding

This paper was supported by the National Natural Science Foundation of China (CL, grant number 82000347; SW, grant number 82070399; JS, grant number 81770374; and YW, grant number 82170362).

## Conflict of Interest

The authors declare that the research was conducted in the absence of any commercial or financial relationships that could be construed as a potential conflict of interest.

## Publisher’s Note

All claims expressed in this article are solely those of the authors and do not necessarily represent those of their affiliated organizations, or those of the publisher, the editors and the reviewers. Any product that may be evaluated in this article, or claim that may be made by its manufacturer, is not guaranteed or endorsed by the publisher.
